# Supplementary material for: TEPEAK: A novel method for identifying and characterizing polymorphic transposable elements in non-model species populations
Source: PLoS Comput Biol. 2026 Jan 6;22(1):e1013122. doi: 10.1371/journal.pcbi.1013122 (PMC12788660; doi:10.1371/journal.pcbi.1013122)
Supplement: S1 Table — (DOCX) [file pcbi.1013122.s001.docx]

| ERE-1 | GGGGCTGGCCCCGTGGCCGAGTGGTTAAGTTCGCGCGCTCCGCTGCAGGCGGCCCAGTGTTTCGTTGGTTCGAATCCTGGGCGCGGACATGGCACTGCTCATCAGACCACGCTGAGGCAGCGTCCCACATGCCACAACTAGAAGAACCCACAACGAAGAATACACAACTATGTACCGGGGGGCTTTGGGGAGAAAAAGGAAAAAATAAAATCTTTAAAAAAAAAA |
| --- | --- |
| ERE-2 | GGGGGCCGGCCCGGTGGCANAGCGGTTAAGTTCGCGCGTTCCGCTTCGGCGGCCCGGGGGTTCACCGGTTCGGATCCCGGGTGCGGACATGGCACCGCTTGGCAAAAGCCATGCTGTGGTAGGCGTCCCACATATAAAGTAGAGGAAGATGGGCACGGATGTTAGCTCAGGGCCAGTCTTCCTCAGCAAAAAGAGGAGGATTGGCAGCAGTTAGCTCAGGGCTAATCTTCCTCAAAAAAAAAAAAAAAA |
| ERE-3 | GGGGCCAGCCCAGTGGCATAGTGGTTAAGTTCATGCGCTCCACTTCAGCGGCCTGGGGTTCGCCGGTTTGGATCCCGGGCACAGACCTACGCACCGCTTATCAAGCCATGCTGTGGCAGGCGTCCCACATATAAAATAGAGGAAGATGGGCACAGATGTTAGCTCAGGGCCAATCTTCCTCAGCAAAAAGAGGAGGATTGGCAGCAGATGTTAGCTCAGGGCTAATCTTCCTCAAAAAAAAAAAAAAAA |
| ERE-4 | GAGCCAGCCCTGATGGCCTAGTGGTTAAAGTTCGGCGCGCTCCGCTTCGGCGGCCCGGGTTCGGTTCCCGGGCGCGGAACCACACCACTCGTCTGTCAGTAGCCATGCTGTGGCGGCGGCTCACATAGAAGAACTAGAAGGACTTACAACTAGAATATACAACTATGTACTGGGGCTTTGGGGAGGAAAAAAAAAAGAGAGAGAGAGGAAGATTGGCAACAGATGTTAGCTCAGGGCGAATCTTTCCCAGCAAAAAAAAAAAAAA |
