# Supplementary material for: TEPEAK: A novel method for identifying and characterizing polymorphic transposable elements in non-model species populations
Source: PLoS Comput Biol. 2026 Jan 6;22(1):e1013122. doi: 10.1371/journal.pcbi.1013122 (PMC12788660; doi:10.1371/journal.pcbi.1013122)
Supplement: S2 Table — (DOCX) [file pcbi.1013122.s002.docx]

| SRA Accession | BREED |
| --- | --- |
| SRR19364562 | QUARTER HORSE |
| SRR19364563 | QUARTER HORSE |
| SRR19364572 | QUARTER HORSE |
| SRR19364590 | QUARTER HORSE |
| SRR19364592 | QUARTER HORSE |
| SRR6474900 | QUARTER HORSE |
| SRR19364560 | QUARTER HORSE |
| SRR19364573 | QUARTER HORSE |
| SRR19364594 | QUARTER HORSE |
| SRR19364595 | QUARTER HORSE |
| SRR19364600 | QUARTER HORSE |
| SRR19364604 | QUARTER HORSE |
| SRR19364606 | QUARTER HORSE |
| SRR19364607 | QUARTER HORSE |
| SRR19364608 | QUARTER HORSE |
| SRR19364609 | QUARTER HORSE |
| SRR19364615 | QUARTER HORSE |
| SRR19364618 | QUARTER HORSE |
| SRR19364633 | QUARTER HORSE |
| SRR19364634 | QUARTER HORSE |
| SRR19364635 | QUARTER HORSE |
| SRR19364637 | QUARTER HORSE |
| SRR19364565 | THOROUGHBRED |
| SRR19364589 | THOROUGHBRED |
| SRR19364593 | THOROUGHBRED |
| SRR515208 | THOROUGHBRED |
| SRR515214 | THOROUGHBRED |
| SRR19364567 | THOROUGHBRED |
| SRR19364580 | THOROUGHBRED |
| SRR19364581 | THOROUGHBRED |
| SRR19364583 | THOROUGHBRED |
| SRR19364584 | THOROUGHBRED |
| SRR19364585 | THOROUGHBRED |
| SRR19364586 | THOROUGHBRED |
| SRR19364587 | THOROUGHBRED |
| SRR19364602 | THOROUGHBRED |
| SRR19364603 | THOROUGHBRED |
| SRR19364605 | THOROUGHBRED |
| SRR19364613 | THOROUGHBRED |
| SRR19364619 | THOROUGHBRED |
| SRR19364621 | THOROUGHBRED |
| SRR19364622 | THOROUGHBRED |
| SRR19364627 | THOROUGHBRED |
| SRR20981598 | ARABIAN |
| SRR20981599 | ARABIAN |
| SRR20981600 | ARABIAN |
| SRR20981611 | ARABIAN |
| SRR6474895 | ARABIAN |
| SRR6474897 | ARABIAN |
| SRR20981622 | ARABIAN |
| SRR6650581 | ARABIAN |
| SRR6650583 | ARABIAN |
| SRR6650662 | ARABIAN |
| SRR6474898 | ARABIAN |
| SRR5469032 | ARABIAN |
| SRR5469031 | ARABIAN |
| SRR5469030 | ARABIAN |
| ERR953412 | ARABIAN |
| SRR4054279 | ARABIAN |
| SRR4054277 | ARABIAN |
| SRR4054242 | ARABIAN |
| SRR3726219 | ARABIAN |
| SRR20981615 | MONGOLIAN |
| SRR20981616 | MONGOLIAN |
| SRR20981617 | MONGOLIAN |
| SRR20981618 | MONGOLIAN |
| SRR6474878 | MONGOLIAN |
| SRR20981619 | MONGOLIAN |
| SRR20981620 | MONGOLIAN |
| SRR20981621 | MONGOLIAN |
| SRR20981623 | MONGOLIAN |
| SRR20981625 | MONGOLIAN |
| SRR20981628 | MONGOLIAN |
| SRR20981629 | MONGOLIAN |
| SRR20981631 | MONGOLIAN |
| SRR20981632 | MONGOLIAN |
| SRR20981634 | MONGOLIAN |
| SRR20981635 | MONGOLIAN |
| SRR12719782 | MONGOLIAN |
| SRR12719781 | MONGOLIAN |
| ERR1527947 | AKHAL-TEKE |
| ERR1527948 | AKHAL-TEKE |
| ERR1527949 | AKHAL-TEKE |
| ERR1527950 | AKHAL-TEKE |
| SRR24676346 | AKHAL-TEKE |
| SRR20981633 | AKHAL-TEKE |
| SRR20981644 | AKHAL-TEKE |
| SRR20981655 | AKHAL-TEKE |
| SRR6474890 | AKHAL-TEKE |
| SRR6474891 | AKHAL-TEKE |
| SRR20981666 | AKHAL-TEKE |
| SRR20981667 | AKHAL-TEKE |
| SRR6474886 | AKHAL-TEKE |
| SRR6599538 | AKHAL-TEKE |
| SRR6650668 | AKHAL-TEKE |
| SRR6474892 | AKHAL-TEKE |
| SRR20981658 | FRIESIAN |
| SRR20981659 | FRIESIAN |
| SRR6474869 | FRIESIAN |
| SRR6474870 | FRIESIAN |
| SRR6474871 | FRIESIAN |
| SRR6474877 | FRIESIAN |
| SRR6474869 | FRIESIAN |
| SRR6650593 | FRIESIAN |
| SRR6650594 | FRIESIAN |
| SRR6650595 | FRIESIAN |
| SRR6650677 | FRIESIAN |
| SRR516118 | JEJU HORSE |
| SRR12719756 | JEJU HORSE |
| SRR12719759 | JEJU HORSE |
| SRR12719761 | JEJU HORSE |
| SRR12719762 | JEJU HORSE |
| SRR12719763 | JEJU HORSE |
| SRR12719764 | JEJU HORSE |
| SRR12719765 | JEJU HORSE |
| SRR12719768 | JEJU HORSE |
| SRR12719770 | JEJU HORSE |
| SRR12719771 | JEJU HORSE |
| SRR527802 | JEJU HORSE |
| SRR20981601 | TIBETAN |
| SRR20981603 | TIBETAN |
| SRR20981604 | TIBETAN |
| SRR20981605 | TIBETAN |
| SRR20981606 | TIBETAN |
| SRR20981607 | TIBETAN |
| SRR8442553 | TIBETAN |
| SRR8442554 | TIBETAN |
| SRR8442557 | TIBETAN |
| SRR8442558 | TIBETAN |
| SRR8442559 | TIBETAN |
| SRR8442562 | TIBETAN |
| SRR8442563 | TIBETAN |
| SRR8442565 | TIBETAN |
| SRR8442566 | TIBETAN |
| SRR8442567 | TIBETAN |
| SRR8442566 | TIBETAN |
| SRR8442567 | TIBETAN |
| SRR8442567 | TIBETAN |
| SRR19364569 | STANDARDBRED |
| SRR19364588 | STANDARDBRED |
| SRR19364591 | STANDARDBRED |
| SRR19364610 | STANDARDBRED |
| SRR19364630 | STANDARDBRED |
| SRR8074196 | STANDARDBRED |
| SRR19364579 | STANDARDBRED |
| SRR2102500 | STANDARDBRED |
| SRR4054239 | STANDARDBRED |
| SRR4054238 | STANDARDBRED |
| SRR892895 | STANDARDBRED |
| SRR892894 | STANDARDBRED |
| SRR892893 | STANDARDBRED |
| SRR891567 | STANDARDBRED |
| SRR891549 | STANDARDBRED |
| SRR891548 | STANDARDBRED |
| SRR891546 | STANDARDBRED |
| SRR891547 | STANDARDBRED |
| SRR19364564 | HANOVERIAN |
| SRR19364597 | HANOVERIAN |
| SRR19364617 | HANOVERIAN |
| SRR19364650 | HANOVERIAN |
| SRR19364659 | HANOVERIAN |
| SRR19364661 | HANOVERIAN |
| SRR2142269 | HANOVERIAN |
| SRR2142163 | HANOVERIAN |
| SRR1046151 | HANOVERIAN |
| ERR978603 | FREIBERGER |
| ERR978604 | FREIBERGER |
| ERR978605 | FREIBERGER |
| ERR978606 | FREIBERGER |
| ERR978608 | FREIBERGER |
| ERR978601 | FREIBERGER |
| ERR978600 | FREIBERGER |
| ERR978607 | FREIBERGER |
| ERR978598 | FREIBERGER |
| ERR978596 | FREIBERGER |
| ERR978602 | FREIBERGER |
| ERR978599 | FREIBERGER |
| ERR978597 | FREIBERGER |
| SRR6607268 | AKHAL-TEKE |
| SRR6474889 | AKHAL-TEKE |
| SRR19364611 | ARABIAN |
| ERR3465846 | ARABIAN |
| ERR3465843 | ARABIAN |
| ERR3465842 | ARABIAN |
| ERR3465841 | ARABIAN |
| ERR3465840 | ARABIAN |
| ERR3465839 | ARABIAN |
| ERR3465838 | ARABIAN |
| ERR3465837 | ARABIAN |
| ERR3465834 | ARABIAN |
| ERR3465845 | ARABIAN |
| ERR3465844 | ARABIAN |
| ERR3465844 | ARABIAN |
| SRR886281 | ARABIAN |
