# Supplementary material for: TEPEAK: A novel method for identifying and characterizing polymorphic transposable elements in non-model species populations
Source: PLoS Comput Biol. 2026 Jan 6;22(1):e1013122. doi: 10.1371/journal.pcbi.1013122 (PMC12788660; doi:10.1371/journal.pcbi.1013122)
Supplement: S1 Protocol — (DOCX) [file pcbi.1013122.s004.docx]

This protocol describes the procedure used to validate TEPEAK’s execution using local (self-hosted) paired-end FASTQ input (“FASTQ mode”). Although the FASTQ files used in this test were obtained from the NCBI Sequence Read Archive (SRA) to provide a reproducible public example, TEPEAK was executed exclusively in FASTQ mode, in which all sequencing reads are supplied as pre-existing local files and no SRA retrieval occurs during pipeline execution.

Paired-end FASTQ files were generated locally for two *Equus caballus* whole-genome sequencing runs (SRR1564422 and SRR1167108) using fasterq-dump with split output (--split-files). This step was performed solely to generate local FASTQ files for testing; TEPEAK itself was not run in SRA input mode.

The horse reference genome assembly GCF_002863925.1 (EquCab3.0) was used for alignment and annotation. The corresponding FASTA and GTF files were downloaded and supplied to TEPEAK as compressed archives, as required by the pipeline.

TEPEAK was executed using the following configuration

species: horse

data_dir: data

output_dir: output

zipped_ref_genome_filepath: data/horse_reference.zip

zipped_gtf_filepath: data/horse_gtf.zip

input_type: fastq

fastq_input:

sample_list: horse_samples.txt

fastq_dir: data/horse/fastq

threads: 8

low: 200

high: 6400

gene: y

run_smoove: false

run_enrichment: false

run_phylogeny: false

tepeak_min_cluster_size: 10

tepeak_percentile_threshold: 75

tepeak_window_size: 50

tepeak_merge_distance: 100

tepeak_pid_threshold: 0.85

tepeak_max_clusters: 50

tepeak_max_sample_seqs: 200

tepeak_dfam_evalue: 1.0

tepeak_dfam_batch_size: 5

tepeak_dfam_delay: 3.0

tepeak_dfam_organism: "Equus caballus"

TEPEAK was executed using the standard Snakemake invocation:

snakemake --configfile config_horse.yaml --cores 8

Successful execution was confirmed by the generation of standard TEPEAK outputs, including per-sample alignments (BAM/BAI files), per-sample insertion call files (VCF), population-level insertion size distributions and peak-finding results, and TE family annotation outputs (Supplementary Table 3).
