# Supplementary material for: TEPEAK: A novel method for identifying and characterizing polymorphic transposable elements in non-model species populations
Source: PLoS Comput Biol. 2026 Jan 6;22(1):e1013122. doi: 10.1371/journal.pcbi.1013122 (PMC12788660; doi:10.1371/journal.pcbi.1013122)
Supplement: S3 Table — (DOCX) [file pcbi.1013122.s005.docx]

| Sample ID | Bases | Total Insertion Calls (>100bp) | Mean Size (bp) |
| --- | --- | --- | --- |
| SRR1564422 | 4.3G | 28 | 1,146 |
| SRR1167108 | 12.7G | 247 | 764 |
