# Supplementary material for: TEPEAK: A novel method for identifying and characterizing polymorphic transposable elements in non-model species populations
Source: PLoS Comput Biol. 2026 Jan 6;22(1):e1013122. doi: 10.1371/journal.pcbi.1013122 (PMC12788660; doi:10.1371/journal.pcbi.1013122)
Supplement: S4 Table — (DOCX) [file pcbi.1013122.s006.docx]

| Merged Insertion Count | 275 |
| --- | --- |
| Mean Insertion Size (bp) | 803 |
