# Supplementary material for: TEPEAK: A novel method for identifying and characterizing polymorphic transposable elements in non-model species populations
Source: PLoS Comput Biol. 2026 Jan 6;22(1):e1013122. doi: 10.1371/journal.pcbi.1013122 (PMC12788660; doi:10.1371/journal.pcbi.1013122)
Supplement: Attachment — Submitted filename: TEPEAK_plos_rebuttal.docx [file pcbi.1013122.s008.docx]

Dear Editor and Reviewers,

We thank the reviewers for their time in submitting constructive feedback on our manuscript. We have addressed each of the comments below. We would like to apologize for any inconvenience reviewers experienced while attempting to run TEPEAK. We acknowledge gaps in our README and uninformative error handling. As a result, we have significantly overhauled the code and documentation to hopefully allow for a more streamlined process. We invite users to file Github issues as needed—user feedback is invaluable for addressing technical concerns and we would be more than happy to further assist in any way.

Thank you for your time,

Devin Burke

**Reviewer #1, Comment 1**

Abstract: The github link provided in the abstract redirects to a different page

### **Response:**

Thank you for pointing this out. The correct link has been added.

*Line 21*

### **Reviewer #1, Comment 2**

Page 5 - "Class II elements move via a “cut-and-paste” mechanism wherein a transposase enzyme directly mediates the joining of the DNA intermediate to its new site" This isn't entirely accurate. Rolling circle transposons are considered Class II but do not use a transposase.

*Line 61*

### **Response:**

Thank you for commenting on this. We agree the statement was overly broad. The wording has been changed to mention “cut-and-paste” and “peel-and-paste” mechanisms.

### **Reviewer #1, Comment 3**

Page 6 - "nearby". This wording assumes the reader is thinking of phylogenetic trees. Probably better to rephrase to "closely related"

### **Response:**

We agree. Closely related is much less ambiguous. The wording has been changed as suggested.

*Line 116*

### **Reviewer #1, Comment 4**

Page 18 - Figure 7. I don't know what this figure is attempting to communicate. Why are the dots spaced along the X axis? What does that spacing mean? Why is half of the red bar not occupied by dots? The explanation in the text doesn't tell us much.

### **Response:**

Thank you for this comment. The x-axis spacing is an artifact of how the sub-ontologies are organized within each of the larger ontology categories. A gap in a certain ontology category simply means that genes for those particular sub-ontologies were not present. However, we agree the gaps were misleading. We updated the figure to eliminate this issue and also updated the figure description to provide a clearer explanation.

*Line 499*

### **Reviewer #1, Comment 5**

Page 19 - Change to "Additionally, this locus..."

### **Response:**

Thank you. The wording has been changed as suggested.

*Line 579*

### **Reviewer #1, Comment 6**

I installed the package and attempted to try it out on some of my own data. This is where several of the comments below arose. The github page provides useful information on how to install the package but that's where the useful information ends. Sooo many questions.

What happens if I want to use the software for a species that does not exist in NCBI or for which there are no SRA entries. Could I use a closely related species? How accurate would it be in that case? There are no explicit instructions on the github page for running my own data. If I have data I haven't uploaded to the SRA, am I out of luck? How do I get those to run? I assume I need to make .bam files based on the github page but how do I get them to run using the software?

### **Response:**

We thank the reviewer for testing TEPEAK. This feedback is greatly appreciated, and we fully acknowledge many gaps in documentation at the time of testing. We have made substantial revisions to the README to address these concerns and have implemented new functionality allowing for custom data input without reliance on SRA. Users can now provide self-hosted FASTQ files directly without additional preprocessing. Regarding the use of closely related species, we cannot make definitive claims about the accuracy of such analyses.

### **Reviewer #1, Comment 7**

The phylogenetics methods section in the manuscript lacks detail. They say the use ML to reconstruct the trees and it looks like the software connects to some tool in EMBL, but what exactly are they using? FastTree, IQTREE? I assume default settings? How is node support determined? Can that be modified? Moreover, why did they mention they can run phylogenetic analyses but not present any trees? Does whatever tree they got actually conform to the current phylogenetic hypotheses for horses?

### **Response:**

Thank you for raising these points. We revised the methodology section to elaborate on how phylogenetic analysis is performed in the workflow. Specifically, we now explicitly state that TEPEAK uses MAFFT for alignment and FastTree for phylogenetic inference. We intentionally did not include phylogenetic trees in our case study because presenting them as evidence would risk implying biological conclusions about TE evolution that require experimental validation. Our decision to include a phylogenetic analysis option in TEPEAK was intended to generalize the tool's capabilities for hypothesis generation and exploration, but this feature did not align with the narrative focus of our case study, which emphasizes the core detection and annotation pipeline.

*Line 248 - 263*

### **Reviewer #1, Comment 8**

Same for the clustering analyses, there are several clustering approaches but they don't mention what they were. How can we replicate the work without this information.

### **Response:**

Thank you for pointing out these oversights. We have expanded the clustering methodology section to address this concern. The revised text now explicitly describes the two-stage clustering approach: (1) adaptive density-based clustering for size-based peak detection using local context-aware thresholds and rolling quantiles, and (2) greedy incremental sequence clustering within size clusters using pairwise sequence identity thresholds. We have included specific algorithmic details, default parameters, alignment methods (Biopython PairwiseAligner with specified scoring schemes), sampling strategies, and consensus selection approaches. This level of detail should enable full replication of the clustering workflow. Additionally, this code is available on our Github for replication concerns.

*Line 248 - 263*

### **Reviewer #1, Comment 9**

In Figure 2, the caption says "The approximate locations where each sample group was sequenced." That wording suggests that they're telling us the location of the lab where the samples were sequenced, but maybe this is a language problem. Shouldn't this refer to collectional localities? The list will need to have more detail. I can read a map but a table with GPS coordinates, cities or at least countries would be helpful. Were they obtained from museums, private collections?

### **Response:**

Thank you for the feedback. We agree that the original figure caption was misleading. Our initial intent was to illustrate both the historical geographic origins of the breeds and the diversity of sequencing locations. However, upon reviewing the available metadata from the public sequence repositories, we found that detailed sample collection locality information (e.g., GPS coordinates, specific collection sites, or institutional sources) was sparse or inconsistent across BioProjects.

To address this concern, we have revised Figure 2 to show only the locations of sequencing institutions from which samples were obtained, with an updated caption that clearly states these locations do not represent sample collection localities. We believe this approach more accurately reflects the available metadata while still illustrating the geographic and experimental diversity of the data sources used in this study.

*Line 312*

**Reviewer #1, Comment 10**
In Figure 5 they have the results of a UMAP analyses, but they don't mention any of that in the methods either. Or is that something that is automatically output by the software?

### **Response:**

Thank you for catching this. The UMAP analysis is not automatically output by the pipeline. A description was added in the methods section for how this analysis was performed relevant to Figure 5B.

*Line 265*

### **Reviewer #1, Comment 11**

Where does the SraRunTable.txt come from? No example is provided. Nore is there any information on how to generate it? Thus, I was immediately dead in the water as far as trying this software out.

### **Response:**

We apologize for the confusion. The README now contains a tutorial for collecting this file and additional instructions for including self-hosted data if desired.

### **Reviewer #1, Comment 12**

How important is the directory structure? It seems that the config file would allow me to set up whatever directory structure I'd like and not use the one suggested at on the github page.

**Response:**

Thank you for noting this issue. We acknowledge that the GitHub documentation was unclear. The README has been overhauled to clarify both directory structure and configuration file settings.

### **Reviewer #2, Comment 1**

1-The authors state that TEPEAK requires a chromosome-level reference assembly. This requirement may limit the tool’s applicability, and I suggest that, if possible, future versions of the pipeline allow genome assemblies at the scaffold level to be used.

### **Response:**

We thank the reviewer for this suggestion. We would like to clarify that TEPEAK can already operate with scaffold-level reference assemblies; there is no technical restriction preventing this. We nonetheless recommend chromosome-level references as the default to maintain accuracy in structural variant and transposable element calling. Scaffold-level assemblies can introduce several sources of error that the tool is not designed to address. We changed the language in the data collection subsection of the methodology section to clarify this point.

*Line 155*

### **Reviewer #2, Comment 2** 2- The manuscript should clarify how the “highest quality” SRA accessions are defined and whether sequencing reads are filtered, trimmed, or otherwise preprocessed prior to analysis.

### **Response:**

Thank you for pointing out this ambiguity. The wording around “highest quality” was removed. The manuscript method section has been updated to specify QC selecting criteria for the horse samples we selected for the case study.

Additionally, the manuscript method section has been updated to mention that no other preprocessing steps were performed outside of the TEPEAK tool for the horse case study.

*Line 322*

### **Reviewer #2, Comment 3** 3- The captions of Figure 1, Table 1, and Figure 2 should be expanded to provide more context and interpretation, ensuring they are fully self-contained.

### **Response:**

Thank you for the feedback. The figure captions have been expanded to be more self-contained.

*Line 140, 312, 318*

### **Reviewer #2, Comment 4** 4- Tool names should be standardized across the manuscript. For example, the text refers to “InSurveyor” in one place and “INSurVeyor” in another. In addition, tool versions should be included where applicable.

### **Response:**

### Thank you for pointing this out. Tool names have been fixed and versions for applicable tools added.

### **Reviewer #3, Comment 1**

- “with over 273,000 different families in the most recent DFAM (3.3)” —> the latest DFAM is 3.9 with 4,121,397 total families (26,279 curated), for a total of 2,784 species

### **Response:**

Thank you. The language has been updated to the latest DFAM version.

*Line 57*

### **Reviewer #3, Comment 2**

- “Class II elements move via a “cut-and-paste” mechanism wherein a transposase enzyme directly mediates the joining of the DNA intermediate to its new site” —> Note that this is not the case ofr RC/Helitrons that are Class II, but use a “peel-and-paste” transposition that effectively copy-and-paste the TE locus.

### **Response:**

Thank you for commenting on this. We agree the statement is overly broad. The wording has been changed to mention “cut-and-paste” and “peel-and-paste” mechanisms.

*Line 61*

### **Reviewer #3, Comment 3**

- “Traditionally, TE families are defined as sequences that share 80% coverage with 80% identity and are thus most often characterized by phylogenetic relationships (although there are families where such characterization is not adhered to)” —> I would argue that biologically speaking, families are a group of homologous sequences expected to originate from a common ancestor locus. The 80-80-80 rule is a useful bioinformatics heuristic but is not grounded in biology.

### **Response:**

Thank you. We agree with your comment. The text has been updated to better reflect both the biological and bioinformatic perspectives.

*Line 65*

### **Reviewer #3, Comment 4**

- “Furthermore, all TEs, to varying degrees, suffer from decay over time which complicates the ability to extract all polymorphic insertions of the same family” —> one could argue that polymorphic families are the most recent one that have colonized a population, and are thus expected to be less degraded than fixed copies.

### **Response:**

Thank you for pointing out this ambiguity. We agree with your comment that polymorphic TE families tend to represent the most recently active elements in a genome and should be less degraded. We recognize that the intent of our original statement was unclear. We revised the text to emphasize that, despite their relative recency, members of even the same TE family can exhibit varying degrees of sequence decay or truncation especially near insertion breakpoints or repetitive contexts. These variations are the real issue with comprehensive recovery of all polymorphic insertions from short read data.

*Line 83*

### **Reviewer #3, Comment 5**

- “Most current methods require the user to provide a list of one or several TE family consensus sequences to detect polymorphic insertions from that family” —> this is correct, though note that tools like Pantera have been developed to circumvent this problem.

### **Response:**

Thank you for pointing out Pantera. We agree that it's a novel tool worth mentioning. We have revised the manuscript to acknowledge Pantera as an exception to the statement about requiring pre-existing TE consensus sequences.

*Line 101*

### **Reviewer #3, Comment 6**

- “leveraging the core idea that elements within the same TE family have similar sizes.” —> I understand the assumption and would think this is correct in most cases, however, I wonder how this could affect an effective detection of LINE-1 elements that are often 5’ truncated and thus do not have a fixed insertion size. (I noticed that some were reported, demonstrating that they can be found, however a discussion on this potential issue would be welcomed)

### **Response:**

Thank you for commenting on this issue. We agree that LINE-1 elements, which are frequently 5′ truncated during retrotransposition, exhibit considerable size heterogeneity that could blur peak boundaries in a purely length-based clustering approach. In TEPEAK, peaks are identified as statistically enriched size *intervals* rather than fixed-length modes, which allows partially truncated LINE-1 insertions to be grouped within broader ranges when they occur at appreciable population frequencies. Nonetheless, we recognize that extreme size heterogeneity—as seen in LINE-1s and other non-LTR retrotransposons—may reduce peak sharpness and thereby complicate detection relative to more size-conserved TE families. We have added a discussion of this limitation to the manuscript.

*Line 615*

### **Reviewer #3, Comment 7**

- Please provide additional details on how, in practice, the consensus built are queried against DFAM
- Explain “pseudo-merged population wide VCF”
- Since I wasn’t able to run the program I could not check, but it seems the program does not output VCF, which would be useful for downstream analyses and comparison purposes.

### **Response:**

Thank you for pointing out these ambiguities.

- A description of the process for building a consensus sequence and its resulting querying against DFAM has been added to the methodology section.
- The “ “pseudo-merged population wide VCF” language has been changed to “merged population-level insertion table summarizing shared and unique loci”.
- We made clarifications to the manuscript and Github README. TEPEAK does produce per-sample VCF files. However TEPEAK does not produce a single merged population-level VCF as this would obscure the biological heterogeneity TEPEAK attempts to unveil in these TE insertions. TEPEAK instead outputs a population-merged insertion table that aggregates all insertion loci detected. We feel this format is more useful for downstream comparative population-structure.

### **Reviewer #3, Comment 8**

- “were locked behind a subscription service” —> though this is described later (GIRI Repbase), the issue should be described at this first occurrence in the MS.

### **Response:**

Thank you for pointing out this issue. We made the appropriate clarification in the manuscript.

*Line 345*

**Reviewer #3, Comment 9**
- Are exonic insertions predicted to be loss of function? If yes, are they expected to contribute to selected traits? Additionally, one could comment that intronic insertion may be functionally important as some can cause alternative splicing.

### **Response:**

Thank you for bringing these issues to our attention. We agree with your assertions, however TEPEAK does not infer functional impact or predict loss-of-function consequences from exonic or intronic insertions; rather, it reports objective overlaps between TE loci and annotated genomic features. While exonic insertions could theoretically disrupt coding sequence integrity, and intronic insertions may influence splicing or expression, such effects cannot be confirmed without experimental validation. Our intent in presenting these results is to describe the distributional patterns of insertions across genomic contexts, which may help prioritize loci for future functional studies.

### **Reviewer #3, Comment 10** Overall the installation appeared successful following the GitHub instructions. However, I strongly recommend to build a complete docker/apptainer version of the whole software to maximize portability. Conda is not always available on HPC, and virtual environment management is not always easy for non specialists. - Suggestion: it would be useful to document how to obtain a compatible SRA table via the NCBI website. - err1 (fixed): “bases” in downloaded SRA run table needed to be changed to “Bases” (uppercase) — the format seem variable. - err2 (fixed): prefetch (src-toolkit) was not in the environment and had to be installed (conda install sra-tools [via bioconda channel]) - Latest error (could not fix): I first thought it was triggered because I didn’t input SRA for bam, but after trying both (SRA/reads and SRA/bams) I obtained the same error. Building DAG of jobs... Using shell: /usr/bin/bash Provided cores: 16 Rules claiming more threads will be scaled down. Job stats: job count -------------------------- ------- align_species 1 all 1 annotate_genes 1 build_histogram_deletions 1 build_histogram_insertions 1 call_insertions_serial 1 dfam_annotate 1 extract_range 1 get_global_vcf 1 smoove_1 3 smoove_2 1 smoove_3 3 smoove_4 1 smoove_global_vcf 1 write_output_genes 1 total 19 Select jobs to execute... /home/reviewer/miniconda3/envs/insurveyor-env/lib/python3.9/site-packages/stopit/__init__.py:10: UserWarning: pkg_resources is deprecated as an API. See<https://setuptools.pypa.io/en/latest/pkg_resources.html>. The pkg_resources package is slated for removal as early as 2025-11-30. Refrain from using this package or pin to Setuptools<81. import pkg_resources [Fri Oct 10 10:16:00 2025] rule align_species: input: data/albo/albo.fa, data/albo/albo.fa.fai, data/albo/albo.fa.amb, data/albo/albo.fa.ann, data/albo/albo.fa.bwt, data/albo/albo.fa.pac, data/albo/[albo.fa.sa](http://albo.fa.sa), data/albo/albo_samples.txt output: data/albo/SRR32381021.bam, data/albo/SRR32381019.bam, data/albo/SRR32381018.bam, data/albo/SRR32381021.bam.bai, data/albo/SRR32381019.bam.bai, data/albo/SRR32381018.bam.bai jobid: 4 reason: Missing output files: data/albo/SRR32381021.bam, data/albo/SRR32381018.bam, data/albo/SRR32381019.bam threads: 16 resources: tmpdir=/tmp Running SRA alignment... 2025-10-10T17:16:00 prefetch.3.2.1: 1) Resolving 'SRR32381021'... 2025-10-10T17:16:01 prefetch.3.2.1: Current preference is set to retrieve SRA Normalized Format files with full base quality scores 2025-10-10T17:16:02 prefetch.3.2.1: 1) Downloading 'SRR32381021'... 2025-10-10T17:16:02 prefetch.3.2.1: SRA Normalized Format file is being retrieved 2025-10-10T17:16:02 prefetch.3.2.1: Downloading via HTTPS... 2025-10-10T17:38:15 prefetch.3.2.1: HTTPS download succeed 2025-10-10T17:38:24 prefetch.3.2.1: 'SRR32381021' is valid: 4426778420 bytes were streamed from 4426769921 2025-10-10T17:38:24 prefetch.3.2.1: 1) 'SRR32381021' was downloaded successfully 2025-10-10T17:38:24 prefetch.3.2.1: 1) Resolving 'SRR32381021's dependencies... 2025-10-10T17:38:24 prefetch.3.2.1: 'SRR32381021' has 0 unresolved dependencies Read 32346618 spots for SRR32381021 Written 32346618 spots for SRR32381021 [Fri Oct 10 10:41:54 2025] Error in rule align_species: jobid: 4 input: data/albo/albo.fa, data/albo/albo.fa.fai, data/albo/albo.fa.amb, data/albo/albo.fa.ann, data/albo/albo.fa.bwt, data/albo/albo.fa.pac, data/albo/[albo.fa.sa](http://albo.fa.sa), data/albo/albo_samples.txt output: data/albo/SRR32381021.bam, data/albo/SRR32381019.bam, data/albo/SRR32381018.bam, data/albo/SRR32381021.bam.bai, data/albo/SRR32381019.bam.bai, data/albo/SRR32381018.bam.bai shell: if [ "sra" = "fastq" ]; then echo "Running custom FASTQ alignment..." bash -x scripts/align_species.sh -s albo -d data/albo -t 16 -f -l data/albo/albo_samples.txt else echo "Running SRA alignment..." bash src/align_species.sh -s albo -d data/albo -t 16 -f data/albo/albo_samples.txt fi (one of the commands exited with non-zero exit code; note that snakemake uses bash strict mode!) Shutting down, this might take some time. Exiting because a job execution failed. Look above for error message Complete log: .snakemake/log/2025-10-10T101559.955648.snakemake.log — Config file: species: albo data_dir: data output_dir: output zipped_ref_genome_filepath: data/GCF_035046485.1_AalbF5_genomic.fna.zip zipped_gtf_filepath: data/GCF_035046485.1_AalbF5_genomic.gtf.zip input_type: sra sra_run: filepath: albo_SraRunInfo.csv number_of_runs: 2 threads: 16 low: 0 high: 10000 gene: y # Include gene annotations SRA table: Run,ReleaseDate,LoadDate,spots,Bases,spots_with_mates,avgLength,size_MB,AssemblyName,download_path,Experiment,LibraryName,LibraryStrategy,LibrarySelection,LibrarySource,LibraryLayout,InsertSize,InsertDev,Platform,Model,SRAStudy,BioProject,Study_Pubmed_id,ProjectID,Sample,BioSample,SampleType,TaxID,ScientificName,SampleName,g1k_pop_code,source,g1k_analysis_group,Subject_ID,Sex,Disease,Tumor,Affection_Status,Analyte_Type,Histological_Type,Body_Site,CenterName,Submission,dbgap_study_accession,Consent,RunHash,ReadHash SRR32628583,2025-07-17 00:24:01,2025-03-10 09:33:13,65729976,19295385812,64307664,293,5183,,<https://sra-downloadb.be-md.ncbi.nlm.nih.gov/sos8/sra-pub-zq-820/SRR032/32628/SRR32628583/SRR32628583.lite.1,SRX27933450,>"=CONCATENATE(A56,""_1"")",WGS,RANDOM,GENOMIC,PAIRED,0,0,ILLUMINA,Illumina NovaSeq 6000,SRP569099,PRJNA1227508,,1227508,SRS24300126,SAMN47286330,simple,7160,Aedes albopictus,Ab21KAHENA104,,,,,,,no,,,,,UNIVERSITY OF FLORIDA,SRA2090678,,public,A9A19922A73CE3C797AF38D2B0D97C71,20C87EE1809F7492FFFDD85E12BA36A7 SRR32628582,2025-07-17 00:23:55,2025-03-10 09:26:26,64654820,19026421115,63104213,294,5087,,<https://sra-downloadb.be-md.ncbi.nlm.nih.gov/sos8/sra-pub-zq-820/SRR032/32628/SRR32628582/SRR32628582.lite.1,SRX27933451,>"=CONCATENATE(A82,""_1"")",WGS,RANDOM,GENOMIC,PAIRED,0,0,ILLUMINA,Illumina NovaSeq 6000,SRP569099,PRJNA1227508,,1227508,SRS24300127,SAMN47286356,simple,7160,Aedes albopictus,Ab21SHIPMN002,,,,,,,no,,,,,UNIVERSITY OF FLORIDA,SRA2090678,,public,103483559021F549A617D88BF6E26236,8B0668B95498D0A4F3A8A9DFC927BC95

### **Response:**

### Thank you for attempting to run TEPEAK. We apologize for the issues and fully acknowledge gaps in the instructions to help set up the tool properly. We have entirely revised the Github README to hopefully resolve any ambiguity. We fully agree that containerization (e.g., via Docker or Apptainer) can enhance portability. However, in practice, containerized workflows can face compatibility challenges on academic (HPC) systems. For this reason, we have prioritized ensuring that TEPEAK’s native Conda-based installation is as robust and accessible as possible, as this approach remains the most widely supported on shared academic computing environments. We plan to explore an Apptainer container in a future release once the native installation and documentation are fully stabilized.

In response to the reviewer’s error log this appears to be a similar issue to a different reviewer stemming from vague guidance in the README and poor error handling in the pipeline. The alignment failed because the documentation did not clearly distinguish between input modes therefore the alignment rule was attempting to re-download already processed runs. The README and example configuration has been revised to make this distinction explicit, and additional error handling has been added to detect misconfigured input types before runtime. We also have included more detailed guidance on the SRA table, implemented case-sensitive parsing in the code, and explicitly added the prefetch library to the default conda environment.

We invite the reviewer (and future users) to submit Github issues if any installation difficulties arise that we can help address.
