# Supplementary material for: TEPEAK: A novel method for identifying and characterizing polymorphic transposable elements in non-model species populations
Source: PLoS Comput Biol. 2026 Jan 6;22(1):e1013122. doi: 10.1371/journal.pcbi.1013122 (PMC12788660; doi:10.1371/journal.pcbi.1013122)
Supplement: Attachment — Submitted filename: TEPEAK_plos_rebuttal_V2.docx [file pcbi.1013122.s009.docx]

Dear Editor and Reviewers,

We thank the editor and reviewers for their careful evaluation of our revised manuscript and for their constructive feedback. We have addressed all remaining comments raised during this round of review and have made targeted revisions to the manuscript, figures, supplementary materials, and documentation where appropriate.

In particular, we have clarified methodological details regarding peak identification, updated figure legends to improve interpretability, and added an experiment demonstrating TEPEAK’s support for local (non-SRA) sequencing data. We believe these changes address the outstanding concerns and further strengthen the clarity and usability of the manuscript.

We appreciate the time and effort invested by the reviewers and hope that the revisions satisfactorily address all points raised.

Sincerely,
Devin Burke

**Reviewer #1, Comment 1**

Lines 64-67 - Cite<https://doi.org/10.1038/nrg2165>, the first publication of this rule.

### **Response:**

Thank you for passing along this paper. We have added its citation to the manuscript.

*Line* 64

**Reviewer #1, Comment 2**

L75 - change to "which do encode protiens for autonomous transposition"

### **Response:**

Thank you for pointing this out. The language has been changed to reflect the suggestion.

*Line 74*

**Reviewer #1, Comment 3**

L321 - I don't understand how these peaks were determined to be significant. There are plenty of peaks surrounding 7000 bp in the second plot in figure 3. More than the four mentioned. What was the threashold for significance? Is that general region the 'mountain' mentioned in line 194 and the four that were listed in the text are the ones that were considered standouts as described in that section? If so, this should be explained in the figure legend.

### **Response:**

Thank you for raising this point. We agree that our original wording did not sufficiently explain how peak positions were identified, which likely caused confusion when interpreting Figure 3, particularly in the 6-7 kb range.

In TEPEAK, insertion-length “peaks” are identified using a local density based procedure with locally defined thresholds. Briefly, insertion counts are aggregated across samples and smoothed using a sliding window. A position is considered elevated when its local density exceeds a locally computed background threshold defined by the 75th percentile within a broader window. Contiguous elevated positions are grouped, and closely spaced groups are merged; within each resulting region, local maxima are reported as representative peak positions.

As a result, regions with heterogeneous but closely spaced insertion lengths such as the ~6-7.5 kb range can give rise to multiple nearby local maxima, all of which are reported. In the revised manuscript and Figure 3 legend, we clarify that the listed values in this range represent closely spaced local maxima within a region of elevated insertion density, rather than implying that each corresponds to a fully independent peak.

*Line 326, 339*

**Reviewer #1, Comment 4**

I'm still disappointed that there's no evidence that a user who plans to use data not in the SRA will succeed. I would very much like to see that the authors ran such an anslysis. It will greatly enhance the usability for other researchers.

### **Response:**

Thank you for raising this point. We agree that explicit evidence demonstrating support for locally supplied data is important. In response, we validated and documented TEPEAK’s support for local (non-SRA) FASTQs.

The full protocol describing this validation run, including configuration details and execution criteria, is provided in Supplementary Protocol S1, and key output summaries are reported in Supplementary Table 3 and 4. This supplement is now referenced in the Data Collection and Preprocessing section of the Methodology.

We hope this addresses the reviewer’s concern and clarifies that TEPEAK can be successfully applied to locally hosted sequencing data. We would be happy to provide any additional clarification if needed.

*Line 155 and Supplementary Information*
